# Bicentric evaluation of employee satisfaction, patient safety and treatment quality: comparing different Health Information System (HIS) solutions

**DOI:** 10.1186/s12913-025-13559-y

**Published:** 2025-11-17

**Authors:** Matthias Brand, Felix Boehm, Udo X. Kaisers, Patrick Fehling, Thomas K. Hoffmann, Nicole Rotter, Sonja Ludwig, Marie-Nicole Theodoraki

**Affiliations:** 1 Department of Otorhinolaryngology, Head and Neck Surgery, Ulm University Medical Center, Ulm, Germany; 2 Ulm University Medical Center, Ulm, Germany; 3 Nursing Management International University Bad Homburg, Bad Homburg, Germany; 4 Department of Otorhinolaryngology, Head and Neck Surgery, Medical Faculty Mannheim, University Medical Centre Mannheim, University of Heidelberg, Mannheim, Germany; 5 Department of Otorhinolaryngology, Head and Neck Surgery, Technical University Munich (TUM), Ismaninger Strasse 22, 81675 Munich, Germany

**Keywords:** Health information systems, Electronic patient file, Digital healthcare systems, Digitalization

## Abstract

**Background:**

Transforming healthcare related work processes through digitization may help to address the growing demands placed on employees by enhancing user satisfaction and may have an impact on patient safety and treatment quality. Hereby, different approaches reaching from multimodal, workflow-specific systems to more uniform and comprehensive formats have been introduced.

**Objectives:**

To date, few comparative studies have examined different Health Information System (HIS) approaches from the perspective of healthcare workers. This study aims to evaluate perceived usability, quality, and compliance of two distinct clinical HIS types.

**Methods:**

In this cross-sectional, bicentric study, we compared a multimodal HIS platform with a variation of different digital systems to a uniform, customized HIS solution that was specifically programmed for Otorhinolaryngology, across two university hospital departments in Germany. A total of *n* = 57 participants (physicians and nurses) completed a 61-item standardized questionnaire. Group differences were analyzed using the Mann-Whitney U test.

**Results:**

Healthcare professionals using the multimodal HIS reported significantly lower overall satisfaction, perceived patient safety and treatment quality compared to those using the customized HIS. The latter system was also rated more positively regarding usability and workflow support. Currently, particularly in Germany, significant efforts are needed across all hierarchical levels to establish a cohesive and clear digital HIS with inter-professional, -institutional, -sectional abilities.

**Conclusion:**

Our study highlights the perceived advantages of a more uniform and customized HIS from the user’s perspective in healthcare, suggesting that these systems could enhance both staff satisfaction and the quality of care.

**Supplementary Information:**

The online version contains supplementary material available at 10.1186/s12913-025-13559-y.

## Introduction

The trend of an increasing proportion of elderly and multimorbid patients [1] is flanked by a shortage of nursing staff [2] with an heavy workload of administrative tasks, which will leave less time for performing the important social tasks of the profession and caring for patients. The digitization of patient records and care curves (Kardex) can counteract the increasing challenges faced by medical staff and provide positive support for employees [3, 4].

Against this backdrop, the bicentric study presented here directly compares a multimodal, workflow-specific hybrid Health Information System (HIS) with a uniform, customized hybrid HIS, assessing employee satisfaction, perceived patient safety, and treatment quality from the perspective of physicians and nurses.

Medical documentation is an essential part of patient care and is regulated by a wide range of legal principles [5]. The documented information is primarily used for medical decision-making [6], as a reminder and “documented memory” [5, 7], as a structure for communication between physician and patient/relatives or in an interdisciplinary setting [7] and ultimately as a planning and organizational aid during the inpatient stay [8]. Secondly, it represents a documentation platform for physicians and nursing staff, which is subject to legal regulations in Germany such as the retention of medical documentation for at least 10 years (§ 10 MBO-Ä), the fulfillment of special security precautions, including data protection when storing electronic files (§ 10 MBO-Ä) and forensic purposes, training and teaching [6]. The general term electronic patient record or computer-based patient record has several definitions, so that further differentiation of this vague term is necessary [6, 9]. The most common classification is the Waegemann stage model [10]. In this model, five hierarchically structured levels are distinguished with different structural criteria and different extents of data information, starting from the computerized paper record (level 1) to the cross-institutional health record (level 5). In this paper, the electronic medical record (level 3) is analyzed.

An electronic patient file (ePF) has several potential advantages. The acquisition and maintenance costs are offset by various medium and long-term opportunities for cost savings (e.g. reduction in paper purchases and space savings due to the elimination of archiving patient files). Despite the high acquisition and maintenance costs of an ePF, an establishment could have a positive long-term effect on the economic situation of the hospital [11–15]. Furthermore, the ePF can be accessed ubiquitously, regardless of time and place [8, 15, 16]. Quick access to all patient data saves a significant amount of time and therefore improves patient care, especially in emergency situations where an overview of the patient’s medical history can be quickly obtained. The ePF enables transparency of entries with regard to completeness, correctness and traceability through user-specific entries, input aids and reminder buttons [6, 15]. Transmission errors due to illegible medication plans are eliminated by digitization. Various search functions increase user-friendliness while providing better access and an improved overview of complex information [6, 8, 16]. A data transfer outside the institution is facilitated by the ePF based on its ability to efficiently and automatically summarize contents and forward them to external bodies [7]. In addition, quality monitoring is possible through various, partially automated data evaluations [7, 15].

A major disadvantage is the wide range of different electronic systems that are not compatible with each other. For example, external documents often have to be scanned into the ePF and cannot be transferred directly from one system to another [6]. The personnel also need to be trained in how to use digital documentation systems. In many cases, however, digital systems exist in parallel with paper-based systems. This complexity is exacerbated by the lack of a standardized cross-institutional, supra-regional or even national IT solution. An IT report survey of the healthcare system in over 1,000 hospitals in Germany in 2017 showed only a medium degree of digitalization in the healthcare system [17].

Therefore, this study compares two hybrid HIS solutions in similarly specialized departments of two German university hospitals, with the aim of identifying system-specific strengths and weaknesses to enhance employee satisfaction, patient safety, and overall treatment quality.

## Methods

### Research design

#### Setting

This bicentric study was conducted in two German university hospitals (site A and site B), each providing maximum-care otorhinolaryngology (ORL) services. Site A uses an electronic patient file (ePF) based on a custom software solution specifically programmed for Otorhinolaryngology (ORL) clinics by a small software company, while site B uses a variation of different digital systems. Both hospitals rely on hybrid documentation combining paper-based Kardex forms with electronic systems. Both hospitals are similar in size with a similar number of physicians and nursing staff employed.

#### Participants

A total of 57 employees took part in the study (site A: 32 [17 physicians, 15 nurses]; site B: 25 [14 physicians, 11 nurses]). Data was collected anonymously in paper form. Informed written consent for the study participation was obtained from all employees.

### Evaluation method of the HIS systems

#### The “HIS Monitor” questionnaire

To systematically evaluate the two different hybrid HIS systems, a questionnaire based on the validated and established questionnaire “Monitoring system for the quality of hospital information systems” (“HIS Monitor”) was employed [18]. The “HIS Monitor” questionnaire was designed by Ammenwerth et al. to evaluate clinical information systems with regard to clinical and administrative tasks and has already been used in other studies [19].

In total, three different types of questions are formulated and answered with three different answer categories according to the four-point Likert-scale [18]:

- “How good…?” Answer option “bad”, “rather bad”, “rather good”, “good”.
- “How appropriate…?” Answer option “inappropriate”, “rather inappropriate”, “rather appropriate”, “appropriate”.
- “How often…?” Answer option “rarely”, “rather rarely”, “rather often”, “often”.

In addition, there is the option to select “Question does not apply to me” for each possible answer to avoid unspecific answers. This was followed by questions about the person, such as occupational group, years of employment in the hospital or experience with computers. Questions on the individual importance of documentation and information-processing tools, such as the importance of documentation for the quality of the work or the time required to use computer-based tools, were asked [20]. In total, this “HIS Monitor” questionnaire consists of 109 questions, 76 of which are aimed for physicians and 77 for nursing staff.

### Structure of the modified “HIS Monitor” questionnaire analyzed here

To ensure feasibility and reduce respondent burden, the original 109-item tool was shortened to 61 items using the following criteria: (i) applicability to both physicians and nurses, (ii) direct relevance to digitized or hybrid documentation processes, and (iii) empirical evidence of impact on user satisfaction or patient-safety outcomes in prior HIS-Monitor studies. Items failing at least two of these criteria were excluded after pilot testing with three clinicians.

Special consideration was taken to ensure that all questions could be answered equally by physicians and nursing staff. Moreover, to preserve the anonymity of the participants, the open personal questions were converted into closed questions (see Appendix A, questions 43–47).

While content validity was upheld by using the validated HIS Monitor as a basis for selection, we recognize that the psychometric characteristics of the abbreviated instrument have not been officially re-validated and should be understood in that context.

### Data analysis

For better statistical analysis, each answer option was converted into a numerical answer. The answer “bad” - “rather bad” - “rather good” - “good” led to the award of two, three, four or five points respectively. The answer option 1 in each question was the statement “Question does not apply to me” and was not considered in the analysis described above. An exception was given for the questions in the “Personal details” section (questions 43–47), where answer option 1 was also awarded one point. In addition, question 43 consists of two answer options (a) nursing staff, c) physician). In each case, 1 or 3 points were awarded. Questions 44 and 45 also consist of two answer options, so that a maximum of 2 points were awarded. A score was created for the general assessment of the stated quality of the hospital information systems depending on the location. Only for the questions regarding the frequency of a negative event or a poorly implemented measure, the scoring was adjusted accordingly: “Frequent” − 2 points, “fairly frequent” − 3 points, “fairly rare” − 4 points, “rare” − 5 points (questions 22, 23, 28, 31, 32, 38).

Average scores were calculated per respondent, and overall group comparisons were conducted based on location (site A vs. B) and profession (physician vs. nurse).

The statistical analysis was conducted using the non-parametric unpaired Mann–Whitney U test. All analyses were performed with GraphPad Prism 10.2.2.

Given the cross-sectional design and the adapted questionnaire, findings should be viewed as exploratory and hypothesis-generating rather than confirmatory.

## Results

### Personal data and IT usage behavior

**Fig. 1:**
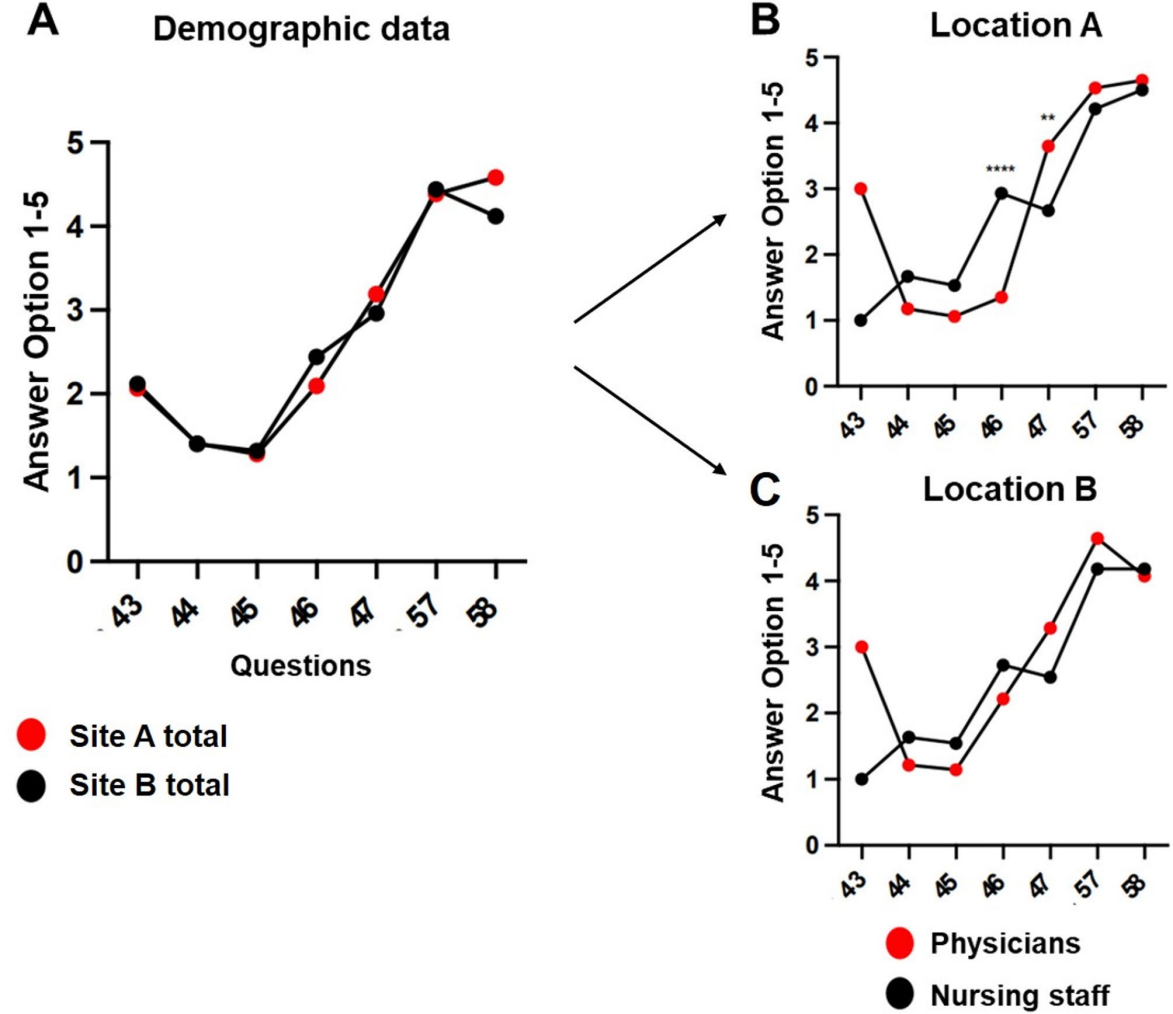
Personal data, questions 43–47 and 57–58 **A**: Presentation of answers given to personal questions from Site A vs. Site B. **B**: Presentation of personal data at Site A (nursing staff vs. physicians) and **C**: at Site B. A significant difference can be seen in questions 46 and 47 at Site A with a significantly higher use of computerized tools among physicians and a significantly higher use of paper-based tools among nurses. ** *p* < 0.005, **** *p* < 0.0001

Personal and professional background data as well as IT experience was collected via questions 43–47, 57 and 58. Table 1 summarizes key demographic and employment data. Figure 1 presents detailed breakdowns by profession and site.

**Table 1:** Information on the interviewees. The study included 32 participants from site A, 17 of whom were physicians and 15 nursing staff. At site B, 25 people took part, including 14 physicians and 11 nurses. The answer option “Question does not apply to me” was ticked 268 times. In 26 cases, open questions and closed questions were not answered, with an accumulation in the participant group of nursing staff on site A. N-numbers in bold, percentage in in brackets

		Nurses Site A	Physicians Site A	Nurses Site B	Physicians Site B	Total
		*n* (%)				
Participants		**15**	**17**	**11**	**14**	**57**
Age	< 45 Years	**7** (47)	**16** (94)	**5** (45)	**12** (86)	**40** (70)
	> 45 Years	**8** (53)	**1** (6)	**6** (55)	**2** (14)	**17** (30)
Duration of employment	< 10 Years	**5** (33)	**14** (82)	**4** (36)	**11** (79)	**34** (60)
	> 10 Years	**10** (67)	**3** (18)	**7** (64)	**3** (21)	**23** (40)
Questions not answered	Closed questions	**23**	**1**	**1**	**1**	**26**
	Open questions	**10**	**7**	**5**	**4**	**26**
Answer option “Question does not apply to me”		**55**	**95**	**56**	**62**	**268**
% Working time with use of paper-based tools	< 20%	**1** (7)	**12** (70)	**0** (0)	**4** (29)	**17** (30)
	20–40%	**3** (20)	**4** (24)	**5** (45,5)	**5** (36)	**17** (30)
	40–60%	**7** (47)	**1** (6)	**4** (36,4)	**3** (21)	**15** (26)
	> 60%	**4** (26)	**0** (0)	**2** (18,1)	**2** (14)	**8** (14)
% Working time with the use of IT-supported tools	< 20%	**0** (0)	**0** (0)	**2** (18,1)	**0** (0)	**2** (4)
	20–40%	**8** (53,3)	**2** (12)	**4** (36,4)	**2** (14)	**16** (28)
	40–60%	**5** (33,3)	**2** (12)	**2** (18,1)	**6** (43)	**15** (26)
	> 60%	**2** (13,3)	**13** (76)	**3** (27,3)	**6** (43)	**24** (42)

Most participants (70%) were younger than 45 years, with physicians being disproportionately younger compared to nurses. Overall, 60% of participants were employed at their current hospital for less than 10 years. Notably, most physicians (80%) had less than 10 years of employment, while over 60% of nurses had more than 10 years (Table 1).

Regarding using digital systems, 76% of physicians at Site A reported using IT-supported tools for over 60% of their work time. Nurses across both sites reported significantly lower IT system usage and greater reliance on paper-based tools (Table 1, Fig. 1A-C).

A total of 268 responses used the option “question does not apply to me,” and 26 questions were left unanswered, primarily by nurses at Site A.

### Evaluation of different HIS approaches

Participants rated the quality of their HIS using a 2–5 scale. Results are summarized in Table 2. Employees of the ORL department at site A (using a customized uniform HIS solution) rated their HIS significantly better on a scale of 2–5 with a median of 3.44 than the ORL department at site B (multimodal HIS platform) with 3.036 (*p* < 0.0001, Table 2). Looking at the individual sites, the nursing staff at site A rated the HIS better than the physicians at site A with a median of 3.655 (median 3.28, *p* = 0.0005). Site B also showed the same evaluation constellation, but with lower median values overall. Looking at the physicians and nurses at the two sites, both groups at site A rated their system significantly better than both professional groups at site B (*p* < 0.0001 for the physicians and *p* = 0.0175 for the nurses, Table 2).

**Table 2:**
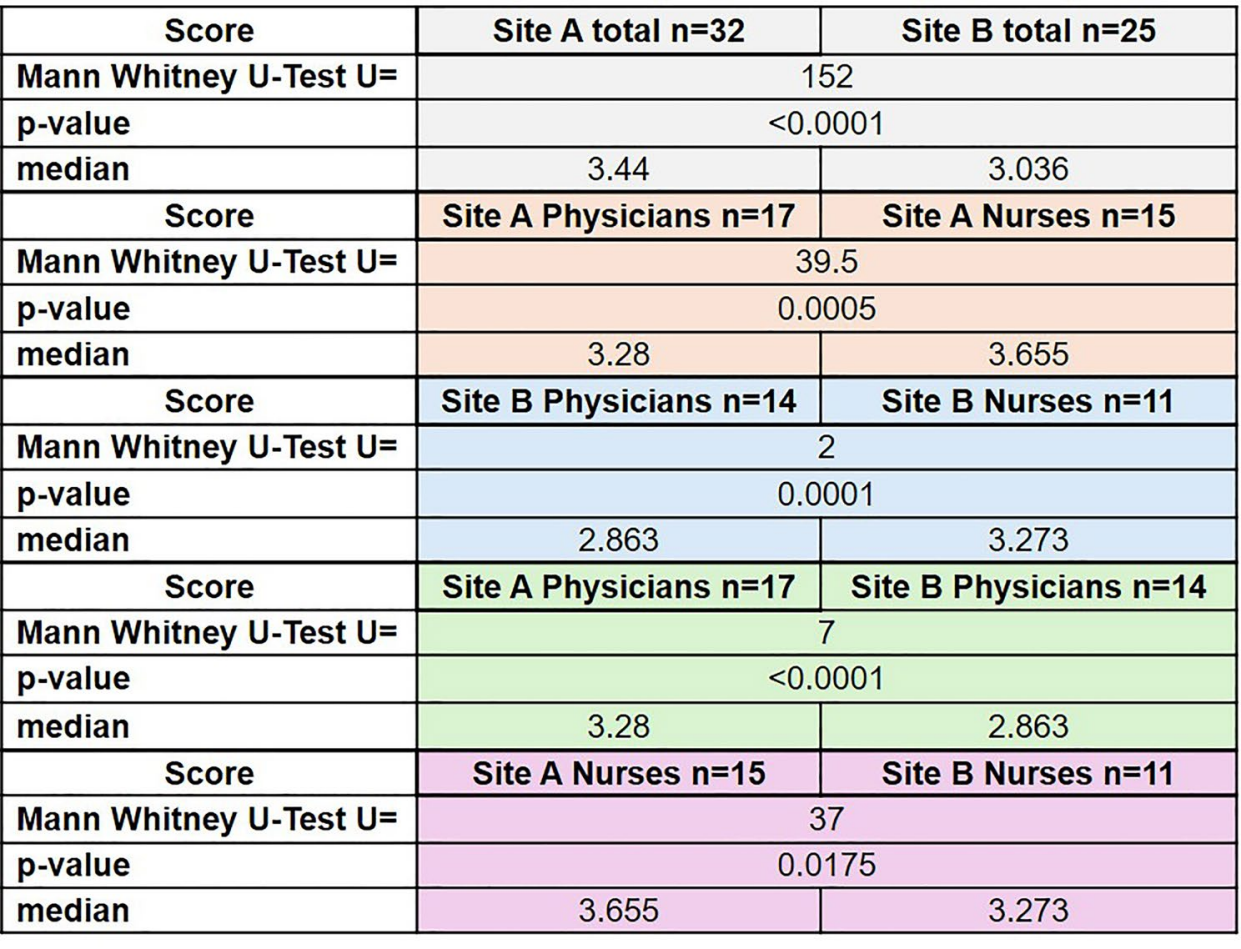
Overall scores for the evaluation of the quality of the clinical information system (HIS, paper and EDP) at site A and site B. Employees of the ENT department at site A rated their HIS significantly better on a scale of 2–5 with a median of 3.44 than the ENT department at site B with 3.036 (*p* < 0.0001). The nursing staff in site A rated the HIS with a median of 3.655 better than the physicians in site A (median 3.28, *p* = 0.0005) with a similar rating constellation in site B. Both professional groups in site A rated their system significantly better than both professional groups in site B (*p* < 0.0001 for physicians and *p* = 0.0175 for nurses)

### Evaluation of documentation and information processing tools

**Fig. 2:**
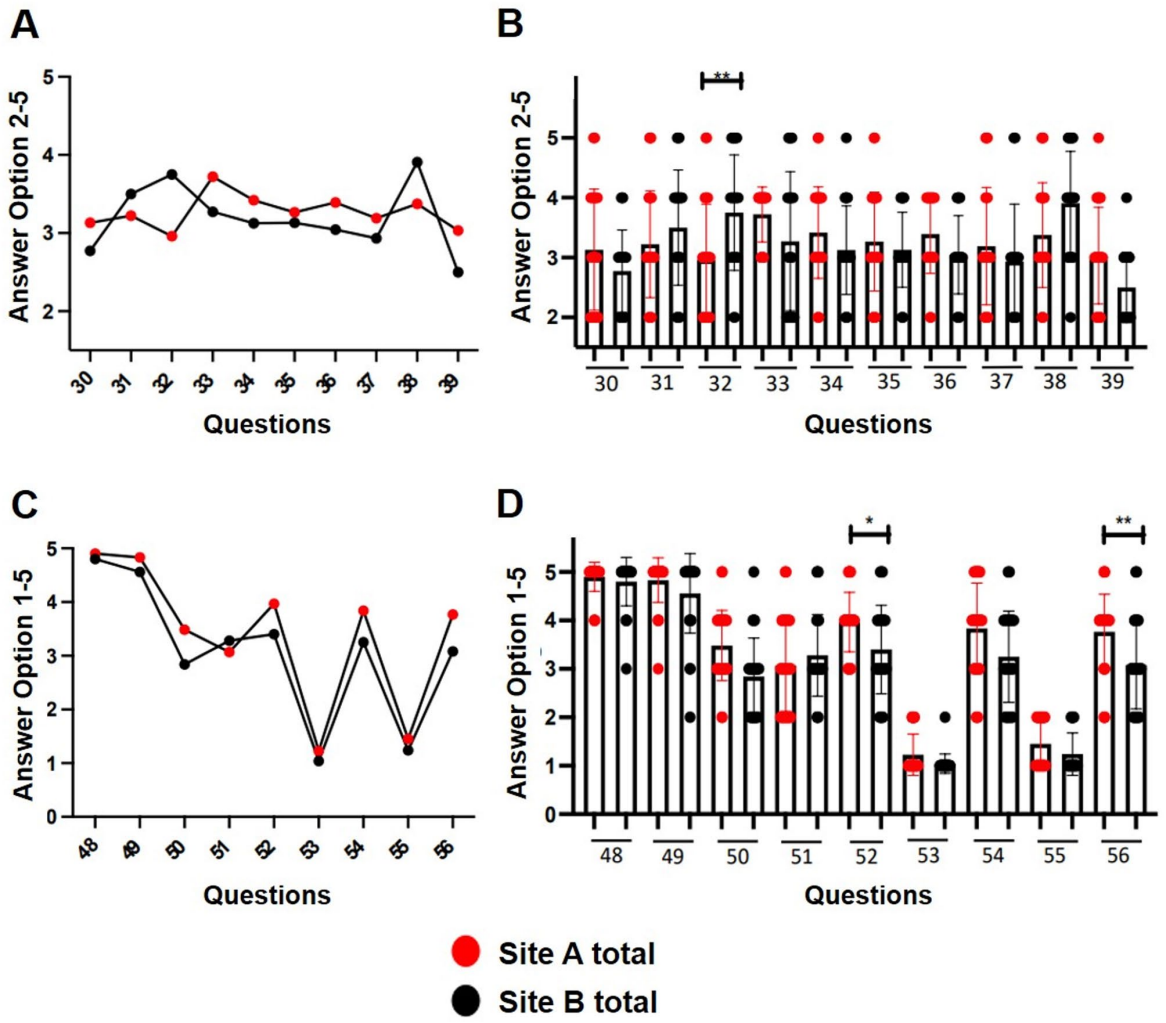
**A + B**, documentation of treatment, questions 30–39, A: comparison of Site A and Site B (mean values), B: graphical representation of the results in box plots with standard deviation of the mean. **p < 0.005. A significant difference was found for question 32: On average, illegible instructions were given more frequently at Site B. **C + D**, information processing tools, questions 48–56, C: comparison of Site A and Site B (mean values), D: graphical representation of the results in box plots with standard deviation of the mean. * *p* > 0.05, **p < 0.005. The employees at Site A feel significantly better supported by IT-based tools in patient care than at Site B (question 52) and report significantly better technical support for questions or problems than at Site B (question 56)

Questions 30–39 and 48–56 assessed documentation quality and IT support in healthcare related work processes (Fig. 2A-D). Illegible handwritten instructions (question 32) were reported more frequently at Site B (*p* < 0.005) (Fig. 2A + B). Both sites indicated rather poor assistance in transferring the outpatient medication list to the care chart (question 30). There were also no significant differences between the sites upon the questions regarding the support in providing an overview of the patient’s upcoming activities (question 35), whether the documentation is complete in accordance with the legal requirements (question 36), how good the support is in transferring the physician’s medication order to the patient chart (question 37) and regarding the perception of time spend for the required documentation (question 39) (Fig. 2A + B). When asked about the frequency of unnecessary multiple documentation of information (question 38), there is a non-significant trend towards increased frequency at site B (Fig. 2A + B).

While both sites expressed dissatisfaction with paper-based tools (question 51), Site A reported significantly better IT-based support (questions 52 and 56, *p* < 0.005) (Fig. 2C + D). Documentation completeness and time demands were rated similarly across sites. In both locations, complete, legible, accurate and prompt patient documentation was considered to be critical for the quality and safety of patient care (questions 48, 49). The support provided by paper-based tools in patient care was uniformly rated as rather poor at both sites (question 51), while the adequacy of the time required for documentation tended to result in higher satisfaction at site A (question 50) (Fig. 2C + D).

### Patient admission and discharge information

**Fig. 3:**
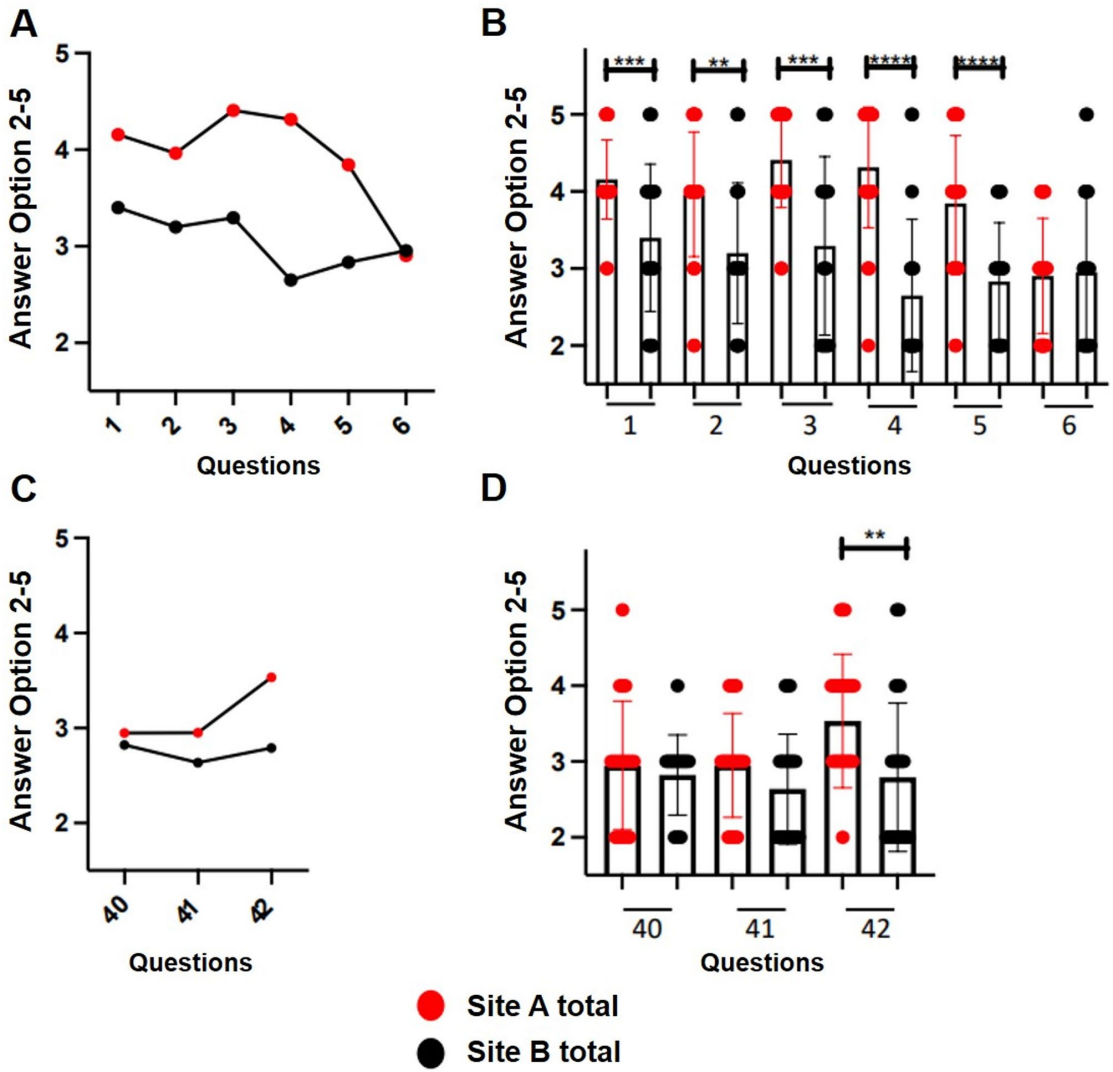
**A + B**, patient admission, questions 1–6: A: comparison of Site A and Site B (mean values), B: graphical representation of the results in box plots with standard deviation of the mean. **p < 0.005, *** *p* < 0.0001, **** *p* < 0.00001. Participants at site A are significantly better supported by the HIS used during patient admission (questions 1–4). **C + D**, patient discharge, questions 40–42, C: comparison of site A and site B (mean values), D: graphical representation of the results in box plots with standard deviation of the mean. **p < 0.005. Both sites indicated rather poor support in creating a discharge care sheet or medication plan and in recognizing incomplete service documentation (questions 40, 41)

Questions 1–6 and 40–42 explored IT support during admission and discharge processes (Fig. 3A-D). The participants at site A are significantly better supported by the HIS used than the staff at site B (questions 1–4) when accessing preliminary information in their own hospital or from external sources, when creating a medical history and when documenting allergies and other risk factors (Fig. 3A + B). According to respondents, access to patient data by an unauthorized person is prevented rather well at site A, while being considered “rather poor” at site B (question 5, *p* < 0.0001). There is no significant difference in the frequency of unreadable data (question 6). While both sites indicate rather poor support in the creation of a discharge care sheet or medication plan and detection of incomplete service documentation (question 40, 41), site A is significantly better supported by the existing HIS in the automatic transfer of diagnoses, anamnesis data, examination findings and physician’s letters (question 42, *p* < 0.005) (Fig. 3C + D).

### Information on the inpatient stay

**Fig. 4:**
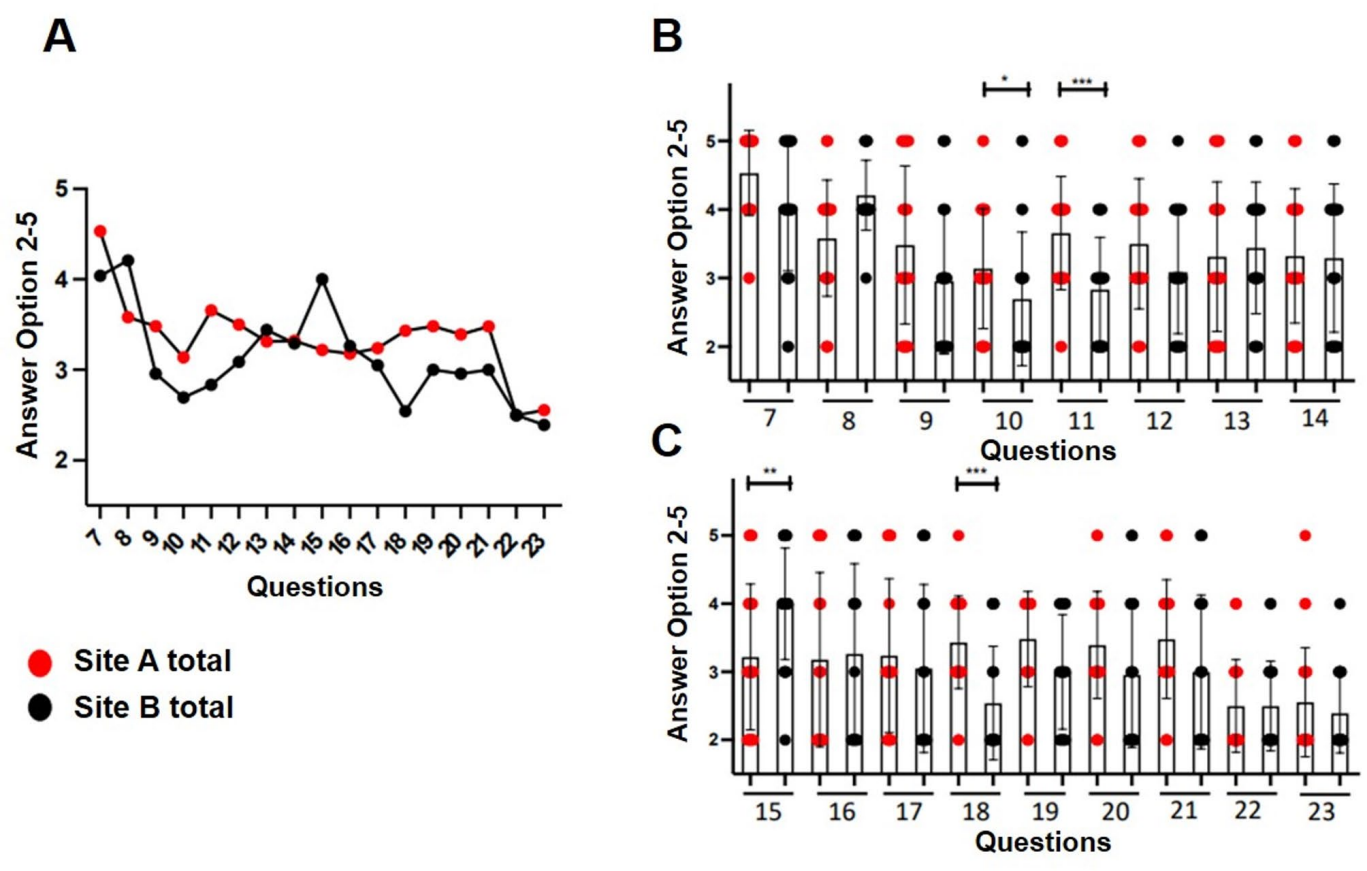
Inpatient stay, questions 7–23: **A**: comparison of Site A and Site B (mean values), **B + C**: graphical representation of the results in box plots with standard deviation of the mean. * *p* < 0.05, **p < 0.005, ***p < 0.0001. Participants in Site A consider the data to be significantly better protected against unauthorized access than those in Site B (question 11). Access to patient data during chief physician rounds is also rated significantly better at Site A than at Site B (question 18)

Seventeen questions (7–23) examined access to relevant patient information during inpatient care (Fig. 4). Site A was rated significantly better for data protection (question 11, *p* < 0.0001) and access during chief physician rounds (question 18, *p* < 0.0001). Both sites reported limited access to appointment data, external results, and nursing forms (Fig. 4). Access to current lab results was rated positively across both locations.

### Information on initiation/change of treatment

**Fig. 5:**
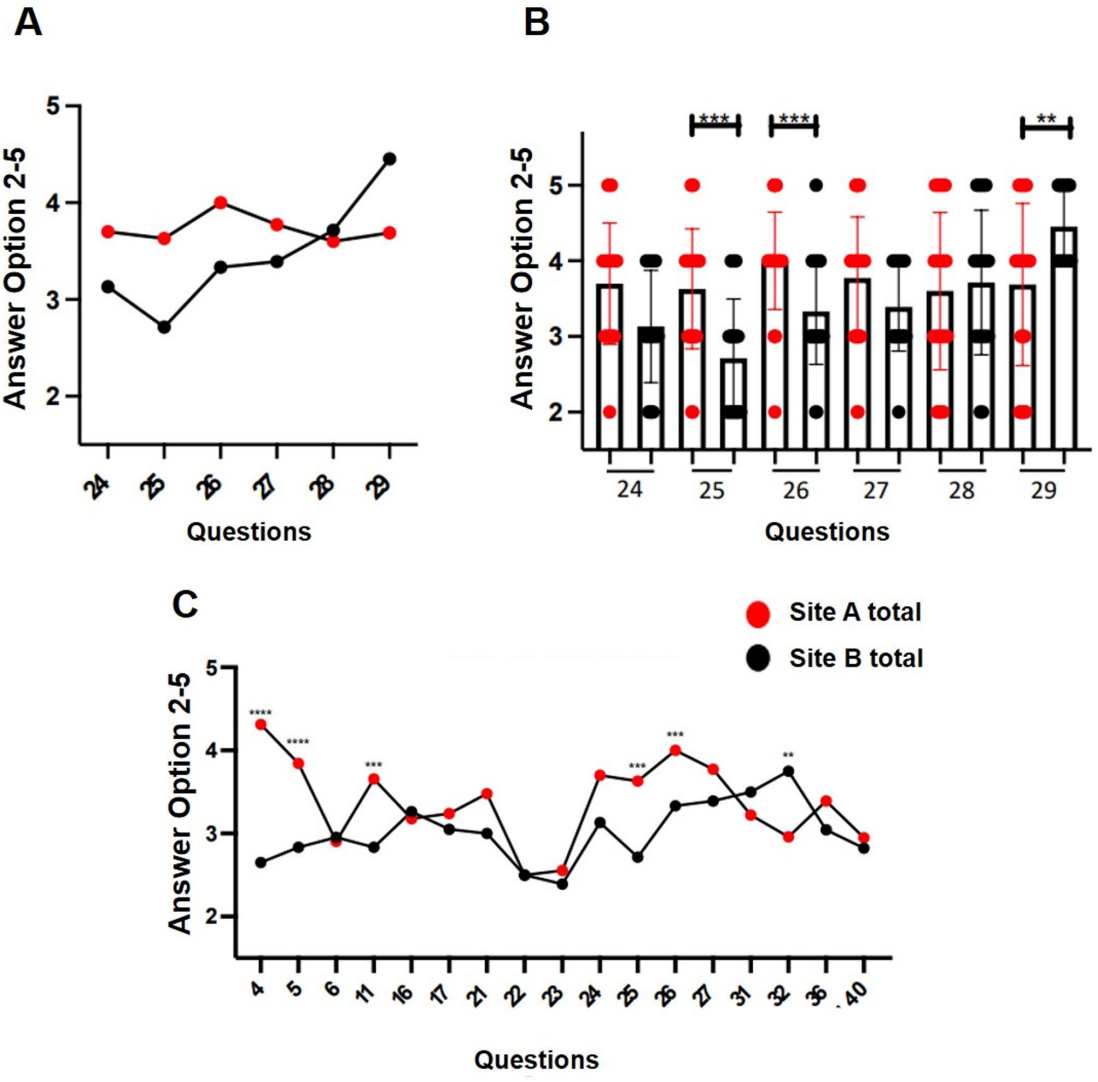
**A-B**: Initiation/change of treatment, questions 24–29: **A**: comparison of Site A and Site B (mean values), **B**: graphical representation of the results in box plots with standard deviation of the mean. **p < 0.005, ***p < 0.0001. Site A reported better provision of information on error prevention and identification of infectious pathogens (questions 25 and 26). **C**: 17 questions on patient safety: Comparison of Site A and Site B (mean values). **p < 0.005, ***p < 0.0001, ****p < 0.00001. In all questions on patient safety, Site A (physicians and nursing staff) gave a significantly better rating than Site B

Questions 24–29 assessed information flow when starting or modifying treatment (Fig. 5A–B). Site A was rated significantly better for error prevention (question 25, *p* < 0.0001), identification of infections (question 26, *p* < 0.0001), and awareness of treatment plan changes (question 24). Illegible author abbreviations (question 29) were reported more frequently at Site B.

### Evaluation of selected questions on patient safety

Seventeen selected questions were evaluated with a focus on patient safety (Fig. 5C). Site A received significantly better ratings for all patient safety-related aspects (*p* < 0.005 to *p* < 0.00001), including legibility of requirements, IT support in identifying risks, and prevention of documentation errors.

### Summary of key findings

Across all thematic areas, respondents at Site A consistently rated their digital infrastructure and process support more favorably than those at Site B. This was evident in system satisfaction (HIS ratings), documentation quality, admission and discharge processes, and IT-based patient care support. While both sites showed limitations in discharge processes and access to certain patient data, Site A demonstrated significantly better performance in areas related to patient safety, technical support, and data accessibility. These patterns were consistent across both professional groups (nursing and medical staff), although nurses at Site A particularly highlighted better system usability. The main results across all thematic blocks are summarized in Table 3.

**Table 3:** Thematic summary of major findings across both study sites

Thematic area	Key findings (Site A vs. Site B)	Significant differences
Personal data and IT usage	Younger workforce among physicians; longer tenure among nurses. Higher IT use at Site A.	Use of IT-based tools: *p* < 0.0001
HIS Evaluation	Overall higher satisfaction with HIS at Site A (especially among nurses).	All subgroups: *p* < 0.05 - *p* < 0.0001
Documentation & Info Processing	Illegible instructions more common at Site B; better IT support at Site A.	Questions 32, 52, 56: *p* < 0.005
Admission & Discharge	HIS support during admission better at Site A; both sites poor in discharge documentation.	Admission: *p* < 0.0001, Discharge: *p* < 0.005
Inpatient Stay	Better data access & privacy at Site A; limited access to some forms at both sites.	Questions 10, 11, 18: *p* < 0.05 - <0.0001
Treatment Change & Safety	Site A better in error prevention and pathogen identification.	Questions 25, 26, 29, 32: *p* < 0.005

## Discussion

### Digitalization in the German healthcare system: structural and cultural barriers

Despite ongoing efforts to promote digitalization, Germany still lacks a standardized, cross-sectoral electronic documentation infrastructure in healthcare. The prevailing bottom-up strategy, favoring local, initiative-driven solutions, has not led to widespread, cohesive improvements. The fragmentation of the healthcare system continue to hinder interoperability and coordinated care [21–23]. These structural limitations contribute to inefficiencies such as redundant diagnostics and communication gaps. However, studies suggest that the implementation of electronic health records (EHRs) can improve both the effectiveness and efficiency of healthcare delivery [24–26]. In addition to the structural problems, patients need to have basic trust in digital solutions. Frequently discussions about data protection and emerging data security problems slowing down the digital progress in Germany [27]. An approach to prevent the creation of a nationwide patchwork of uncoordinated individual solutions could be for legislators to establish national guidelines that could, for example, consist of binding solutions with uniform objectives, a fixed budget and timetable, clear data protection regulations, data structures and standards for interoperability.

### Comparative evaluation of HIS functionality and user experience

In this study, notable differences in HIS evaluations were observed between the two otolaryngology departments, although both operated under similar general conditions. Given the limited sample, these findings should be regarded as context-specific rather than broadly generalizable. Physicians and nurses at site A, where a specialized customized electronic patient file (ePF) is in use, consistently rated IT-based tools, process integration, and patient safety features more favorably than participants at site B. The integration of clinical functions into a single digital system and the customized HIS approach at site A were perceived more positively by participants. These results suggest a potential link between tailored digital solutions and higher user satisfaction, but further research across different settings is needed to confirm such associations.

### Professional perspectives: diverging needs of physicians and nurses

An exploratory finding of this study is the difference in HIS evaluation between professional groups. Physicians were generally less satisfied with the systems than nurses, particularly at site B. While nurses at both sites continue to rely heavily on the paper-based Kardex system, physicians expressed greater concern regarding legibility, duplication of documentation, and limited integration of clinical processes. While these differences are in line with prior research [10, 28–31] the limited scope of our study does not allow firm conclusions but rather points to trends that merit further exploration.

### Role of local IT support, training and customized software

The higher acceptance of IT tools at site A may in part be related to more reliable local IT support. However, due to the restricted sample, this interpretation should be considered with caution. A dedicated IT team and tailored training offerings likely contributed to participants’ positive assessment of digital documentation and safety mechanisms, such as allergy alerts and real-time visibility of infectious risks. These safety features, largely absent at site B, were perceived to contribute directly to error prevention during prescribing and clinical decision-making. Prior studies have similarly highlighted the role of system usability and local support in fostering acceptance of EHRs [6, 8, 19, 32]. 

### Data security: perceptions and practices

The participants at site B rated data protection significantly worse than the participants at site A. Enhanced data protection can be achieved with the adoption of electronic files because access is only possible with an individual, employee-specific password [33]. On the other hand, there are concerns in the literature that the use of electronic files in particular can represent a lack of data protection, for example due to weak password protection or employees forgetting to log out on mobile devices, with the risk of unauthorized access to patient data [11, 34]. For this reason, users at site A are automatically logged off after a certain idle time and are asked to change their password at regular intervals. When using a paper-based file, access is theoretically possible for any unauthorized person who is in the vicinity of an unattended file.

These findings suggest that both system design and user behavior must be addressed to ensure secure data handling.

### Workflow challenges and process-specific insights

The questionnaire responses also indicate persistent challenges across both sites. Problems were reported in both hospitals regarding the transfer of the patient’s own medication plan and access to patient information during various everyday situations. Due to the possibility of using a mobile application of the ePF on a tablet PC-based, portable form site A, the criticism here was not as strong as at site B. The care chart (Kardex), which is similarly structured in both hospitals, is indicated as bulky and thus causes more difficulties in availability in all necessary situations.

Participants at site A criticized the lack of information about changes to the treatment plan. The frequency of illegibility of an abbreviation was also criticized more strongly at site A than at site B. Regarding the avoidance of medication errors, allergic complications or the identification of infectious pathogens, the respondents at site A felt significantly better supported than the participants at site B. Warning symbols light up in the ePF at site A as soon as a patient is selected or appears in the ward overview (see Figure S1), and entries are marked directly with a physician’s abbreviation. These safety mechanisms at site B are less clear and not always comprehensible. Digital provision of the medication file can potentially prevent transmission errors [35]. Patient safety may be increased by the use of electronic patient records compared to paper records, as important preliminary information for treatment decisions is more readily available and a quicker overview can be obtained in emergency situations [6, 8, 19]. However, our findings alone cannot establish causality.

In open question 61, the respondents at site B wish for a new system that integrates all different software tools used at their site, so that only one software program needs to be employed. The transferability of findings in physician’s letters, the provision of digital files forward rounds as well as the integration of warning functions for allergies or infections are also desired. In general, this describes the situation with the ePF already present at site A. Both professional groups would like to see the abolition of paper-based documentation and the creation of a standardized documentation system for the nursing staff and physicians.

### Limitations of the study

This study has several limitations that should be considered when interpreting the findings. First, the sample size was relatively small and restricted to two ORL departments, which limits the generalizability of the results to other clinical settings or disciplines. Second, no power analysis was performed prior to data collection, so the statistical power of the study remains uncertain. Furthermore, the study relied solely on quantitative survey data, without triangulation through qualitative interviews, observational methods, or document analysis, which may have limited the depth of insight into participants’ experiences. Third, the cross-sectional design provides a snapshot of user perceptions but does not allow for conclusions about causality or changes over time.

Moreover, although the HIS questionnaire was adapted to the specific needs of this study, no formal validation or reliability testing was conducted for the modified version.

Another limitation lies in the unexpectedly high number of “does not apply to me” responses (*n* = 268). Although the questionnaire was shortened and simplified during the pretesting phase, some participants still found it too long or difficult to understand. This may have led to item nonresponse due to fatigue, confusion, or a lack of perceived relevance. Additionally, we did not assess participants’ prior familiarity with key terminology, such as “electronic medical record,” “computerized paper record,” or “hybrid documentation system”, which may have affected the validity of certain responses. This should be considered when interpreting the findings and addressed in future studies through more comprehensive pretesting and terminology clarification.

### Policy implications and health care digitalization in an international comparison

From a policy perspective, these results support calls for a more unified strategy to promote interoperable, user-centered documentation tools. However, recommendations should be framed cautiously: the findings are context-specific, and broader generalizations should account for variations in infrastructure, culture, and institutional readiness.

The current situation regarding the implementation, acceptance, and use of digital hospital information systems in Germany is characterized by a significant catch-up process. Nevertheless, Germany continues to lag behind countries such as the United States, France, Italy, the United Kingdom, and China in international comparison [21, 36–41]. 

By contrast, the United States is a global leader in the widespread adoption and use of electronic health records (EHRs), characterized by high interoperability and strong patient engagement, supported by substantial financial incentives and regulatory frameworks while having significantly less data protection [42, 43]. France, following initial challenges with the *Dossier Médical Partagé* (DMP), has now achieved broad usage of electronic patient records, facilitated by national strategies and incentive programs. In Italy, certain regions, such as Lombardy, serve as frontrunners, with highly interoperable systems and successful standard integration [44, 45]. In the United Kingdom, digitalization is nearly complete in the outpatient sector, while efforts in the inpatient sector continue to focus on integration and interoperability. China has achieved an exceptionally high EHR adoption rate (> 85%) through state-led programs, with a strong emphasis on integration into regional networks and electronic data exchange [21, 36–41, 46]. 

In summary, Germany remains behind in international comparison but is gradually advancing due to targeted policy measures and investments. The most pressing challenges continue to be interoperability, cross-sectoral integration, and sustainable acceptance in everyday clinical practice [21, 36–41]. 

Future digital initiatives in Germany should therefore balance top-down standardization with local flexibility, ensuring systems are interoperable but adaptable to clinical workflows. Given the growing complexity of digital documentation, particular attention should be paid to professional training and change management strategies [3, 47, 48]. The HIS Monitor used in this study served as a valuable screening tool to highlight user-reported deficits, but future research should complement such surveys with workflow analysis and outcome-based evaluation.

### Interpretative summary: key findings

Taken together, four central findings emerge:

1. System integration seems to play an important role: The presence of a unified digital platform (e.g., the ePF) was associated in this sample with more positive evaluations of documentation quality, workflow efficiency, and patient safety.
2. Professional groups may have differing needs and expectations: Physicians prioritize completeness, legibility, and real-time access, while nurses emphasize ease of use and bedside documentation compatibility.
3. Organizational support may be an important factor: Local IT teams and tailored training were reported by participants as supportive and could contribute to higher user satisfaction.
4. Caution is required in implementation and policy-making: While digitalization offers clear benefits, its success depends on system design, user engagement, and institutional commitment. Given our limited sample, these conclusions should be viewed as indicative rather than definitive.

## Summary

The healthcare system relies on the exchange of information and data, along with efficient communication. This study investigated how electronic patient records affect the quality of HIS in two top-tier otorhinolaryngology (ORL) hospitals, focusing on the viewpoints of physicians and nursing personnel. A quantitative research study was carried out utilizing a revised HIS questionnaire.

At both sites, nursing documentation was conducted using a paper-based Kardex, whereas medical documentation differed: site A used a customized, ORL-specific electronic patient file (ePF), while site B relied on a fragmented mix of digital tools. Staff at site A evaluated their HIS more positively across multiple dimensions, including technical support, documentation quality, and perceived patient safety. In contrast, the use of multiple non-integrated systems at site B was associated with higher levels of dissatisfaction.

A key finding was the divergence in satisfaction between professional groups: physicians expressed greater dissatisfaction with paper-based workflows, while nursing staff were more critical of the limited integration of their documentation into digital systems. Meanwhile, particular nurses voiced a need for improvements in processes such as medication ordering or therapy guidance. These needs highlight the possible advantages of adopting a digital documentation system tailored specifically for nursing.

Our data suggest that a well-designed, department-specific ePF, as implemented at site A, can contribute to increased satisfaction, reduced documentation workload, and improved access to patient data. In this context, the ePF used at site A may serve as a promising example of a tailored, functional HIS solution. Looking ahead, medium-term efforts should focus on improving interdisciplinary in-house interoperability to support treatment transparency and coordination. In the long term, cross-institutional electronic health records could enable seamless data sharing between different healthcare sectors. For this to succeed, supportive infrastructure, clear responsibilities, and sufficient training are essential. However, several limitations must be acknowledged when interpreting the results. First, the study included a relatively small sample from two specialist departments, which limits generalizability. Second, although the HIS questionnaire was adapted and pretested, it was not formally revalidated. The high number of “does not apply to me” responses suggests that some items may have been misunderstood or perceived as irrelevant. Third, the study lacked triangulation with qualitative or observational data, and we did not assess participants’ understanding of key terminology, such as the difference between “electronic health record” and “hybrid system.” These aspects should be addressed in future research.

Overall, our results support the idea that focused, user-centered digital initiatives, based on actual clinical requirements, can enhance both employee satisfaction and the quality of patient care.

## Supplementary Information

Below is the link to the electronic supplementary material.

Supplementary Material 1: Figure S1: A: Drug allergies marked in the ePA at Site A. If a medication allergy is indicated on admission, the electronic file is marked with a red exclamation mark (red pill), which is always visible when the file is opened. The allergy is noted by hand in the Kardex and color-coded. B: Biohazard warning hazard sign in the ePA at Site A. If an infectious pathogen is detected and marked in a patient, the ePF is color-coded

## Data Availability

The data that support the findings of this study are available from the corresponding author upon reasonable request.

## Abbreviations

HIS: Health information system
ORL: Otorhinolaryngology
MBO-Ä: Model professional code for physicians
ePF: Electronic patient file
IT: Information technology
