# Supplementary material for: Bicentric evaluation of employee satisfaction, patient safety and treatment quality: comparing different Health Information System (HIS) solutions
Source: BMC Health Serv Res. 2025 Nov 17;25:1470. doi: 10.1186/s12913-025-13559-y (PMC12621393; doi:10.1186/s12913-025-13559-y)
Supplement: Supplementary file 1 — Supplementary Material 1: Figure S1: A: Drug allergies marked in the ePA at Site A. If a medication allergy is indicated on admission, the electronic file is marked with a red exclamation mark (red pill), which is always visible when the file is opened. The allergy is noted by hand in the Kardex and color-coded. B: Biohazard warning hazard sign in the ePA at Site A. If an infectious pathogen is detected and marked in a patient, the ePF is color-coded [file 12913_2025_13559_MOESM1_ESM.pptx]

## Slide 1
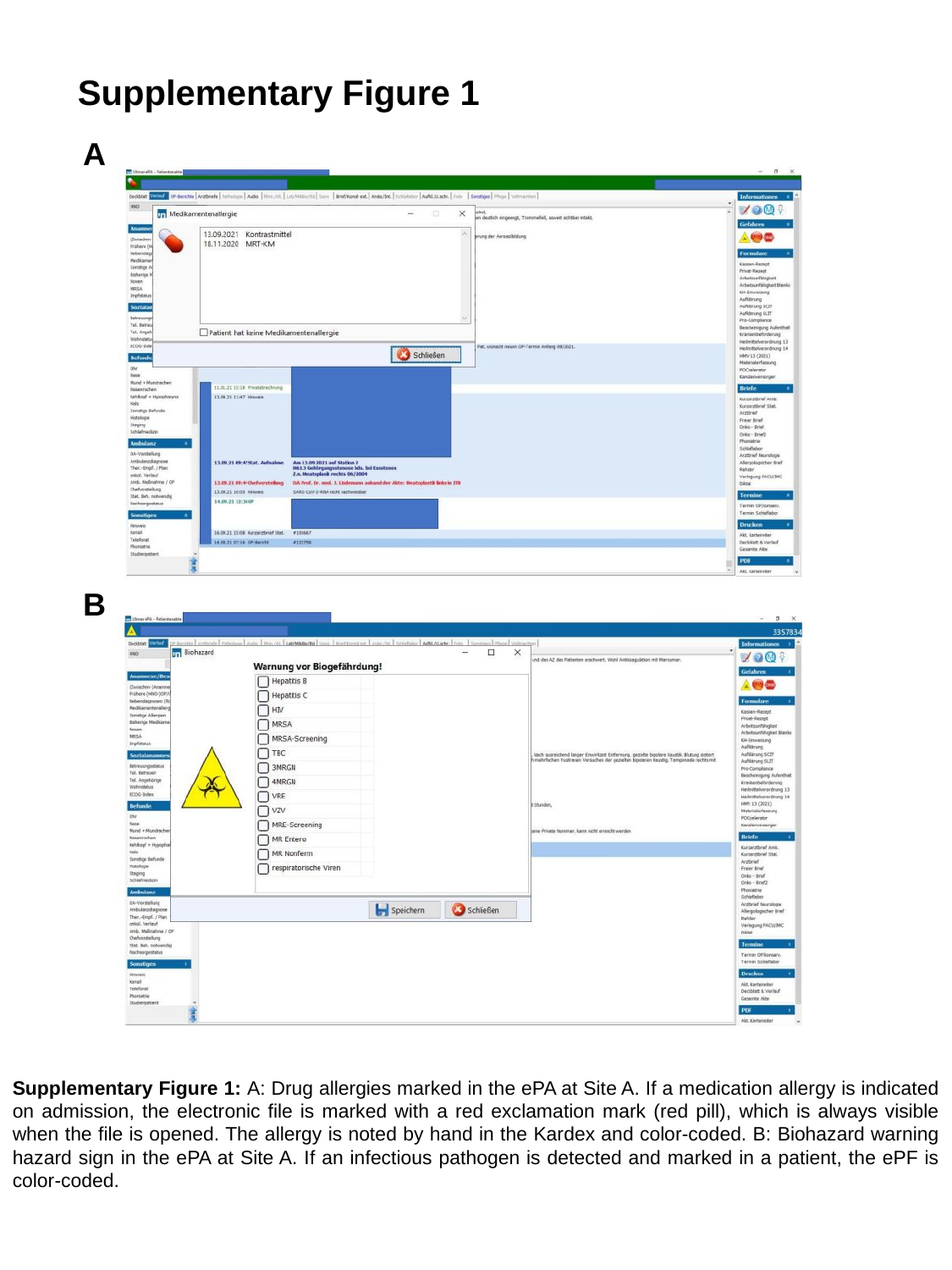

Supplementary Figure 1
A
B
Supplementary Figure 1: A: Drug allergies marked in the ePA at Site A. If a medication allergy is indicated on admission, the electronic file is marked with a red exclamation mark (red pill), which is always visible when the file is opened. The allergy is noted by hand in the Kardex and color-coded. B: Biohazard warning hazard sign in the ePA at Site A. If an infectious pathogen is detected and marked in a patient, the ePF is color-coded.
